# Carbon Monoxide Induced PPARγ SUMOylation and UCP2 Block Inflammatory Gene Expression in Macrophages

**DOI:** 10.1371/journal.pone.0026376

**Published:** 2011-10-25

**Authors:** Arvand Haschemi, Beek Yoke Chin, Markus Jeitler, Harald Esterbauer, Oswald Wagner, Martin Bilban, Leo E. Otterbein

**Affiliations:** 1 Department of Laboratory Medicine, Medical University of Vienna, Vienna, Austria; 2 Department of Surgery, Beth Israel Deaconess Medical Center, Harvard Medical School, Boston, Massachusetts, United States of America; Chinese University of Hong Kong, Hong Kong

## Abstract

Carbon monoxide (CO) dampens pro-inflammatory responses in a peroxisome proliferator-activated receptor-γ (PPARγ) and p38 mitogen-activated protein kinase (MAPK) dependent manner. Previously, we demonstrated that CO inhibits lipopolysaccharide (LPS)-induced expression of the proinflammatory early growth response-1 (Egr-1) transcription factor in macrophages via activation of PPARγ. Here, we further characterize the molecular mechanisms by which CO modulates the activity of PPARγ and Egr-1 repression. We demonstrate that CO enhances SUMOylation of PPARγ which we find was attributed to mitochondrial ROS generation. Ectopic expression of a SUMOylation-defective PPARγ-K365R mutant partially abolished CO-mediated suppression of LPS-induced Egr-1 promoter activity. Expression of a PPARγ-K77R mutant did not impair the effect of CO. In addition to PPARγ SUMOylation, CO-activated p38 MAPK was responsible for Egr-1 repression. Blocking both CO-induced PPARγ SUMOylation and p38 activation, completely reversed the effects of CO on inflammatory gene expression. In primary macrophages isolated form C57/BL6 male mice, we identify mitochondrial ROS formation by CO as the upstream trigger for the observed effects on Egr-1 in part through uncoupling protein 2 (UCP2). Macrophages derived from bone marrow isolated from Ucp2 gene Knock-Out C57/BL6 mice (*Ucp2* ^−/−^), produced significantly less ROS with CO exposure versus wild-type macrophages. Moreover, absence of UCP2 resulted in a complete loss of CO mediated Egr-1 repression. Collectively, these results indentify p38 activation, PPARγ-SUMOylation and ROS formation via UCP2 as a cooperative system by which CO impacts the inflammatory response.

## Introduction

Carbon monoxide (CO) arises physiologically in cells during the oxidative catabolism of heme by heme oxygenase (HO) enzymes [1]. CO administered exogenously to cells or animals at low concentrations exerts anti-inflammatory effects in a number of preclinical models [2], [3], [4], [5], [6], [7]. Previously, we demonstrated that CO suppresses LPS-induced expression of Egr-1, a key transcription factor in the generation of the inflammatory response [8] via activation of the nuclear hormone receptor PPARγ [9] (reviewed in [1], [10]). Since CO treatment alone does not result in generation of PPARγ ligands, activity-modulating posttranslational modifications of PPARγ in response to CO may occur, such as phosphorylation or SUMOylation. Recent genetic, molecular, and biochemical studies support the idea that PPARγ inhibits inflammatory gene expression in activated macrophages through a SUMOylation-dependent pathway [11], [12], [13]. SUMOylation is a protein modification involving a covalent conjugation of the polypeptide SUMO to lysine residues of target proteins [14]. SUMOylation has emerged as a significant regulatory mechanism in cell physiology as it relates to processes such as inflammation. SUMO modification can affect, in a target-specific fashion, a protein's subcellular and subnuclear localization as well as its ability to interact with other proteins and/or its activity in transcriptional processes [14], [15], [16]. SUMOylation of PPARγ targets it to co-repressor complexes that are bound to inflammatory response gene promoters and, as such prevents their signal-dependent clearance that is normally a prerequisite for transcriptional activation. As a consequence, genes remain in a repressed state [11].

Accumulating evidence suggests that CO, particularly in macrophages, signals in part through a brief but marked generation of mitochondrial-derived ROS (reviewed in [1] and [17]). Importantly, this transitory ROS burst by CO exposure selectively augments LPS-induced p38 phosphorylation as well as PPARγ activation; both events are fundamental for CO to suppress proinflammatory gene expression in macrophages [2], [3], [9], [18]. The source of mitochondrial-derived ROS involves the family of uncoupling proteins (UCP) [19]. There are three known UCP proteins; UCP1, 2 and 3, each postulated to be involved in regulating respiration, thermogenesis, fatty acid oxidation and metabolism [20], [21]. UCP can modulate excessive ROS generation originating from complexes I and III of the mitochondrial respiratory chain [22]. Conversely, ROS such as superoxide was demonstrated to induce UCP2-mediated proton leak in cultures of isolated mitochondria [25] suggesting the plausibility that CO-induced transitory ROS burst may indirectly influence UCP function. Nevertheless, the detailed molecular mechanism leading to augmented ROS in response to CO remain unclear.

In this study, we extend our previous observations and have identified CO-induced PPARγ-SUMOylation as a novel mechanism by which CO regulates PPARγ and thereby the acute inflammatory response in macrophages. We show for the first time that Ucp2 is indeed critical for CO to enhance mitochondrial ROS formation. Moreover, ROS mediated p38 activation and PPARγ-SUMOylation by CO cooperate to repress inflammatory gene expression.

## Materials and Methods

### Cell culture

RAW 264.7 mouse peritoneal macrophages and human embryonic kidney 293FT cells from ATCC (Rockville, MD) were cultured in DMEM containing 10% FBS and 50 µg/ml gentamicin. Cells were exposed to CO (250 ppm) or air as previously described [2] for indicated times. Following pretreatment of RAW264.7 cells with CO, LPS (10 ng/ml *E. coli* serotype O127:B8; Sigma, St Louis, MO) was added as indicated in the text. Ucp2 gene KO mice (*Ucp2* ^−*/*−^) were a kind gift or Dr. Brad Lowell (Division of Endocrinology, Department of Medicine, Beth Israel Deaconess Medical Center and Harvard Medical School, Boston, MA, USA) and described previously [23]. Bone Marrow Derived Macrophages (BMDM) were isolated from C57/BL6 or *Ucp2* ^−*/*−^ mice (Jackson Laboratory, Bar Harbor, Maine, USA) and differentiated as previously described [24]. All animal work was approved by the Institutional Animal Care and Use Committee at the Beth Israel Deaconess Medical Center (Protocol number: 090-2008).

### SUMOylation assays

As SUMOylation assay, we preferably employed the well established *in vivo* (in cells) SUMO-model than the common *in vitro* (cell-free) model. This *in vivo* SUMO-model was previously described and is widely accepted for SUMOylation research [11]–[13], [15] Briefly, 293FT cells were transfected in 10 cm dishes with HA-tagged wild-type PPARγ (kindly provided by B. M. Spiegelman, Dana-Farber Cancer Institute & Department of Cell Biology, Harvard Medical School, Boston, MA, USA), Myc-tagged SUMO-1 (kindly provided by C.K. Glass; Department of Medicine, University of California, San Diego, La Jolla, CA) and Protein inhibitor of activated STAT, 1 (PIAS1; kindly provided by Dr. Tony Hollenberg, Department of Endocrinology, Beth Israel Deaconess Medical Center, Harvard University, Boston, MA, USA). 36 hours later, cells were treated with air, CO (250 ppm) or Rosiglitazone (5 µM; BioMol, Plymouth Meeting, PA) or N-Acetylcysteine (NAC; Sigma) for 16 hrs. Thereafter, cells were washed twice with NEM-PBS buffer (20 mM N-ethylmaleimide, Sigma, in PBS) and lysed in lysis buffer containing 0.1% SDS, 0.5% deoxycholate, 0.5% TritonX-100, 1 mM EDTA, 20 mM N-ethylmaleimide, 20 mM Tris-HCl pH 7.4, 150 mM NaCl and complete protease inhibitor cocktail (Roche). 250 µg cell lysates was immunoprecipitated for 4 hours with either polyclonal anti-PPARγ, or SUMO-1 antibody (Abcam, Cambridge, MA, USA) coupled to Protein G sepharose (Sigma) and washed four times in lysis buffer. Immunoprecipitates were resolved by SDS–PAGE.

### CO exposure

Cells were exposed to CO, using a bioactive gas-controlling system custom designed and built by Biospherix. To achieve a concentration of 250 ppm, CO (Lifegas) was mixed with 5% CO2/20.8% O2/74% N2 and controlled by Watflow Anafaze software.

### ROS measurement

Mitochondrial superoxide generation was assessed using MitoSOX Red superoxide indicator (Invitrogen, Carlsbad, CA). MitoSOX Red reagent is live-cell permeable and selectively targeted to the mitochondria. Once in the mitochondria, MitoSOX Red reagent is oxidized by superoxide, the predominant ROS in mitochondria, and exhibits red fluorescence. Briefly, bone marrow derived macrophages (BMDM) were incubated with 5 µM MitoSOX for 30 min at 37°C prior to harvest. The cells were then washed, exposed to air or CO for 5–120 min, resuspended in FACS buffer (PBS+1%FBS), and analyzed on a fluorescent activated cell sorter (FACS), (BD Biosciences, San Diego, CA). Forward and side scatter gates were set to include cells but to exclude debris and remaining unbound particles. Excitation/Emission was set at 488/580 nm (MitoSOX). Each experimental set was performed 3 times, and the data analyzed with CellQuest™ software (BD Biosciences, San Diego, CA, USA).

### SDS-PAGE and Western blotting

Cell lysates were separated by SDS-PAGE and blotted onto Polyvinylidene fluoride (PVDF)-membranes (GE Healthcare, Amersham, Buckinghamshire, UK) as described previously [9]. After blocking, blots were incubated overnight (4°C) with primary rabbit anti Egr-1, or mouse anti HA-tag (Santa Cruz Biotechnology). Membranes were then washed in TBST (Tris-Buffered Saline+0.05% Tween-20) and visualized using HRP-conjugated antibody against rabbit or mouse IgG and the ECL reagents (Amersham, Piscataway, NJ), per manufacturer's instructions. To confirm equal loading, blots were re-probed with mouse monoclonal antibody targeting β-actin (Abcam Inc.). Densitometry analysis was done with Quantity One (Bio-Rad).

### Real-time PCR

Total RNA was extracted from RAW 264.7 cells using the Rneasy mini kit (Qiagen) according to manufacturer's protocol. 1 µg total RNA was reverse transcribed into cDNA by MMLV enzyme (Promega, Mannheim, Germany) with random hexamers (1 µg/µg total RNA). The reaction mixture was incubated at 37°C for 45 minutes followed by 15 min at 45°C and 20 min at 70°C. All PCRs were performed using the SYBR Geen kit (BioRad, Hercules, CA, USA). Primers for selected genes were designed via the Primer3 software (http://frodo.wi.mit.edu/cgi-bin/primer3/primer3_www.cgi) and are as follows: iNOS (forward: 5′-AATCTTGGAGCGAGTTGTGG-3′; reverse: 5′-CAGGAAGTAGGTGAGGGCTTG-3′); Rplp0 (forward: 5′-GCCAATAAGGTGCCAGCTGCTG-3′; reverse: 5′- GAAGGAGGTCTTCTCGGGTCCTAG-5′). Using the ABI Prism 7700 sequence detection system (PE Applied Biosystems, Warrington, UK), PCR cycling conditions were as follows: initial denaturation at 95°C for 10 min, followed by 40 cycles at 94°C for 30 seconds, 60°C for 15 seconds and 72°C for 30 seconds and a 10 minutes terminal incubation at 72°C. Sequence Detector Software (SDS version 1.6.3, PE Applied Biosystems) was used to extract the PCR data, which were then exported to Excel (Microsoft, Redmond, WA) for further analyses. Expression of target genes were normalized to Arp expression levels. Data were analyzed according to the 2^−ΔΔCT^ method.

### Vector construction, transient transfection & Egr-1 luciferase reporter assays

The murine Egr-1 promoter/luciferase construct (pGL3-Egr1) containing nt −617 to −7 from the 5′upstream region was cloned in the luciferase vector pGL3 (Promega, Wallisellen, Switzerland) with XhoI and HINDIII into the corresponding sites. RAW 264.7 cells were seeded into 48well plates and transfected 1 day thereafter at 1.0 µg/well with consisting of 0.6 µg of either pcDNA3.1 (Invitrogen, Carlsbad, CA), PPARγ-K77R, PPARγK365R (kindly provided by C.K. Glass; Department of Medicine, University of California, San Diego, La Jolla, CA) along with 0.4 µg pGL3-Egr1 and 0.02 µg phRL-TK (Promega) using Superfect reagent (Qiagen) according to the manufacturer's instructions. The mutants are characterized by lysine-to-arginine amino acid substitutions (K77R and K365R), which impair ligand-dependent PPARγ transactivation or transrepression, respectively, and were successfully tested for expression at the protein level [11]. 24 hrs later, cells were treated with CO, Rosiglitazone (BioMol, Plymouth Meeting, PA), SB203580 (25 µM; Calbiochem), U0126 (25 µM, Cell Signaling, Boston, MA) or vehicle (Dimethyl sulfoxide). After 6 hrs, protein extracts were assessed for luciferase activity (Promega).

### Statistical Analysis

All data unless otherwise indicated are shown as mean values ± standard error of the mean (SEM) and tested statistical using two-tailed Student's t test or ANOVA. All figures and statistical analyses were generated using GraphPad Prism 4. p<0.05 was considered to indicate statistical significance.

## Results

### CO treatment increases SUMOylation of PPARγ dependent on ROS

**Figure 1:**
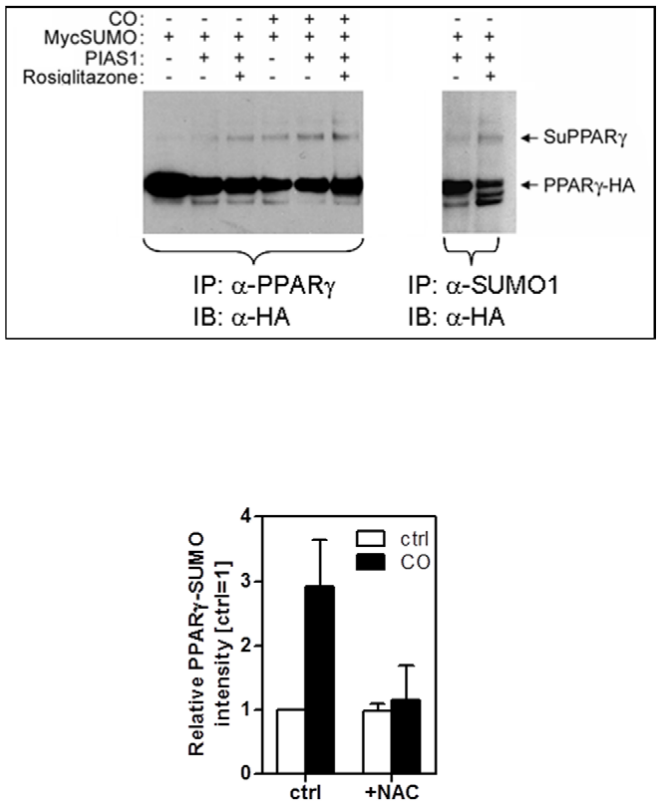
CO treatment enhances SUMOylation of PPARγ. (**A**) 293FT cells were transfected in 10 cm dishes with mixtures of expression plasmids, consisting of 2.5 µg HA-PPARγ, 5.5 µg myc-SUMO-1, and 2.0 µg of PIAS1 as indicated. 24 hours after transfection, cells were incubated for 12–18 hours with Rosiglitazone and/or CO (250 ppm) followed by immunoprecipitation of PPARγ (left panel) or SUMO-1 (right panel) from cell lysates and immunoblotting for HA-tag. The western blots are representative of three independent experiments. (**B**) 293FT cells transfected with the expression plasmids for HA-PPARγ, myc-SUMO-1 and PIAS1 were incubated for 12–18 hours with or without CO (250 ppm) in the presence or absence of N-Acetylcysteine (10 mM). SUMOylated PPARγ was calculated as the ratio of SUMOylated PPARγ versus total PPARγ form pull-down assays. Shown are mean +/− SEM densitometry values of three independent experiments.

In order to understand the mechanism by which CO activates PPARγ we tested for PPARγ SUMOylation. As a model we reverted to the standard SUMO-assay in HEK293FT cells. HA-tagged PPARγ, Myc-tagged SUMO1, PIAS1 or pcDNA3.1 (as a control vector) were efficiently co-transfected in cells and subsequently the SUMO-target PPARγ was affinity-purified with an antibody directed against PPARγ and further subjected to western blot analysis. By anti-HA antibody incubation the presence of HA-tagged PPARγ was verified in all immunoprecipitates (**Fig. 1A**). When PIAS-1 was co-expressed, additional slower migrating PPARγ bands with a molecular mass of approximately 140–150 kDa were detected (indicated by ‘SuPPARγ’ in **Fig. 1A**). Treatment with Rosiglitazone, a selective ligand of PPARγ known to induce SUMOylation, increased the intensity of SuPPARγ. Treatment with CO also increased this high molecular weight PPARγ (**Fig. 1A**). To ensure that the slower migrating bands indeed represented PPARγ conjugated to SUMO-1 we repeated immuno-precipitations with an anti-SUMO1 antibody instead of anti-PPARγ to capture SUMOylated proteins. Probing these SUMO-precipitates using an anti-HA detection antibody resulted in the very same high molecular weight bands as seen for PPARγ immuno-precipitation and thereby ensured specific detection of SUMOylated PPARγ by this method (**Fig.1A** right panel). Because CO induces a transient burst of mitochondrial ROS that leads to increased PPARγ expression [9], we hypothesized that CO-induced ROS may also be responsible for PPARγ-SUMOylation. In the presence of the ROS scavenger N-Acetyl-Cysteine (NAC), CO exposure no longer resulted in PPARγ-SUMOylation (**Fig. 1B**). This identified PPARγ SUMOylation by CO as a process dependent on ROS formation.

### SUMOylation of PPARγ by CO is required for suppression of proinflammatory genes

**Figure 2:**
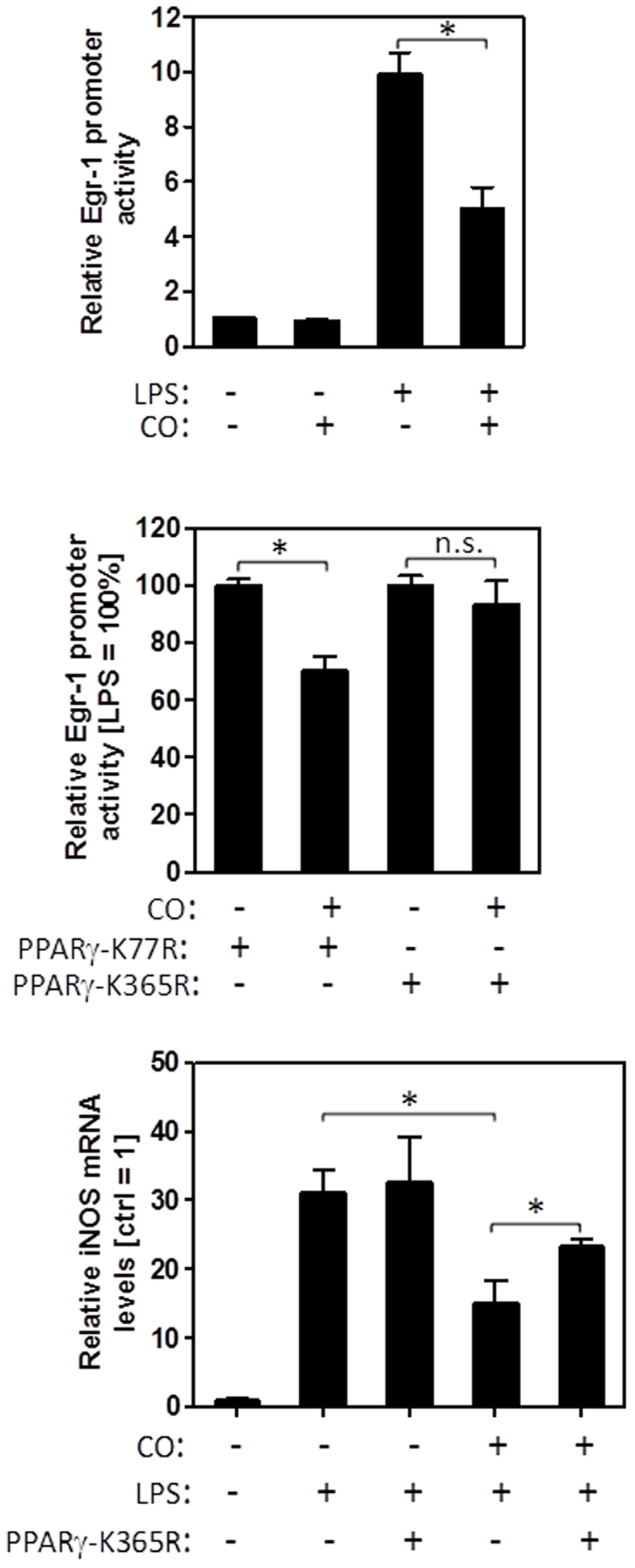
CO requires SUMOylated PPARγ to suppress proinflammatory gene expression. (**A**) RAW 264.7 cells were transiently transfected with pcDNA3.1 along with a firefly luciferase reporter construct under the control of the murine Egr-1 promoter (pGL3-Egr1) and pRL-TK as internal control followed by treatment with LPS (10 ng/ml) in the absence or presence of CO (250 ppm). Note that LPS provoked a strong and robust Egr-1 luciferase signal which was inhibited in the presence of CO. (**B**) RAW 264.7 cells were transiently transfected with a SUMOylation-defective PPARγ mutants (PPARγ-K365R or PPARγ-K77R) or pCDNA3.1 control vector followed by treatment with LPS in the absence or presence of CO (250 ppm). Total proteins were harvested at 6 hr post induction and dual luciferase activities determined. The value of firefly luciferase was normalized by the rennila luciferase to generate the relative luciferase activity. (C) RAW 264.7 cells were transiently transfected with a SUMOylation-defective PPARγ mutant (PPARγ-K365R) or pCDNA3.1 control vector ( = ctrl) followed by treatment with LPS in the absence or presence of CO (250 ppm). RNA was extracted and iNOS mRNA levels (normalized to the Rplp0 gene) measured by real-time PCR. Bars represent mean values ± SEM of three independent experiments.

Next, we determined the functional significance of CO-induced PPARγ SUMOylation by introducing PPARγ mutants that cannot be SUMOylated. As a readout we established a transrepression assay based on LPS induced Egr-1 promotor activation. RAW 264.7 macrophages were co-tranfected either with PPARγ-K77R, PPARγ-K365 or empty vector along with an Egr-1 luciferase reporter before cells were stimulated with 10 ng/ml LPS, in the absence or presence of CO (250 ppm). LPS treatment resulted in a strong upregulation of the Egr-1 promoter, which was inhibited by CO exposure (**Fig. 2A**). Ectopic expression of PPARγ-K77R in RAW cells did not affect the inhibitory potential of CO on Egr-1 expression in LPS-activated cells. However, inhibition was partially reversed in RAW264.7 cells expressing PPARγ-K365R, suggesting a causative role of CO-mediated PPARγ SUMOylation at amino acid residue 365, but not 77, in reducing Egr-1 activation (**Fig. 2B**). In order to explore whether these results were limited to Egr-1 or reflected a widespread requirement for PPARγ-SUMOylation in regulating inflammatory genes, we measured iNOS mRNA expression. As previously reported, LPS treatment caused strong upregulation of iNOS mRNA, which was blunted in the presence of a CO (**Fig. 2C**). This CO-mediated iNOS inhibition was partially reversed in PPARγ-K365R expressing cells (**Fig. 2C**). These results show for the first time that CO is able to functionally regulate PPARγ activity by promoting K365 SUMOylation.

### CO-mediated PPARγ SUMOylation and p38 MAPK cooperate to Suppress Egr-1

**Figure 3:**
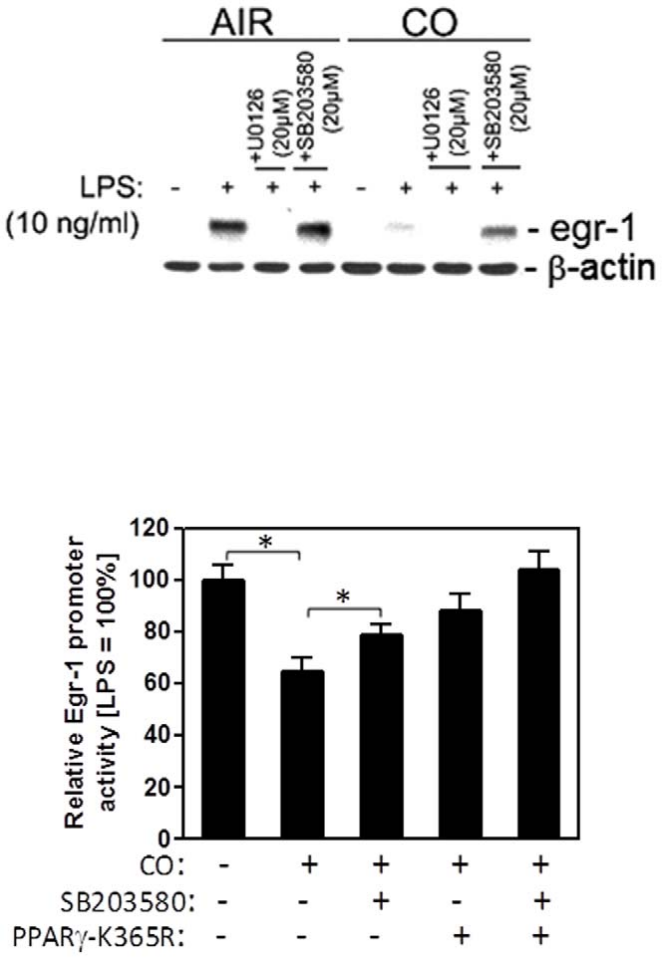
CO-mediated PPARγ-SUMOylation and p38MAPK activity cooperate for full suppression of LPS-induced Egr-1 promoter activity. (A) RAW 264.7 cells were pretreated with 250 ppm CO or Air for 3 hours, followed by a further 30 min incubation with MAPK inhibitors for p38 (SB203580) or ERK1/2 (U0126) prior to stimulation with LPS (10 ng/ml) for 1 hour. Egr-1 protein expression was analyzed by western blotting and β-actin was used as a loading control. The western blot is representative of three independent experiments. (B) RAW 264.7 cells were transiently transfected with PPARγ-K365R or pcDNA3.1 control vector along with pGL3-Egr1 and phRL-Tk. Thereafter, RAW 264.7 cells were pretreated with 250 ppm CO or Air for 3 hours, followed by a further 30 min incubation with SB203580 or vehicle (DMSO) prior to stimulation with LPS (10 ng/ml). Total proteins were harvested at 6 hr postinduction and dual luciferase activities determined. The value of firefly luciferase was normalized by the rennila luciferase to generate the relative luciferase activity. Bars represent mean values ± SEM of three independent experiments (^*^P<0.05).

Previously, we demonstrated that CO induces mitochondrial ROS and thereby hyperphosphorylation of p38 MAPK, which is required for suppression of LPS-induced TNFα secretion in RAW 264.7 macrophages (2, 18). In respect to Egr-1 expression, p38 hyperactivation by CO has not been evaluated as a possible regulator. To test for a possible role for p38 in CO-mediated suppression of LPS-induced Egr-1 expression we examined LPS-activated macrophages treated with air or CO in the presence of pharmacologic inhibitors for MAPKs. LPS treatment resulted in a robust increase in Egr-1 protein expression, which was blunted when cells were pretreated with CO (**Fig. 3A**). Pharmacologic blockade of ERK-1/2 with U0219 completely blocked Egr-1 expression irrespective of CO treatment. p38 inhibition partially reversed CO-mediated ablation of Egr-1 expression, whereas no effect of p38 blockade was observed in control cells. This clearly indicated CO-induced p38 activation as an additional mechanism as to how CO exposure results in Egr-1 repression. To interrogate the nature of the cooperative effect between PPARγ-SUMO and p38 mediated Egr-1 repression, we individually and jointly blocked the respective pathways in the presence of CO. We measured Egr-1 expression in LPS-activated and CO exposed macrophages either pretreated with a specific inhibitor of p38 MAPK (p38α, p38β and p38β2) activity, SB203580 and/or transfected with PPARγ-K365R mutant. RAW 264.7 cells treated with LPS substantially upregulated Egr-1 promoter activity, which was blocked when cells were pretreated with CO (**Fig. 3B**). SB203580 treatment, to block p38, as well as transfection PPARγ-K365R mutant only partially reversed the CO effect. However, p38 MAPK inhibition in PPARγ-K365 expressing cells fully ablated repression of Egr-1 luciferase activity (**Fig. 3B**). This identified CO mediated Egr-1 repression as a process dependent on prior p38 activation and PPARγ SUMOylation.

### CO regulates ROS via UCP2 to suppress LPS-activated Egr-1 expression

**Figure 4:**
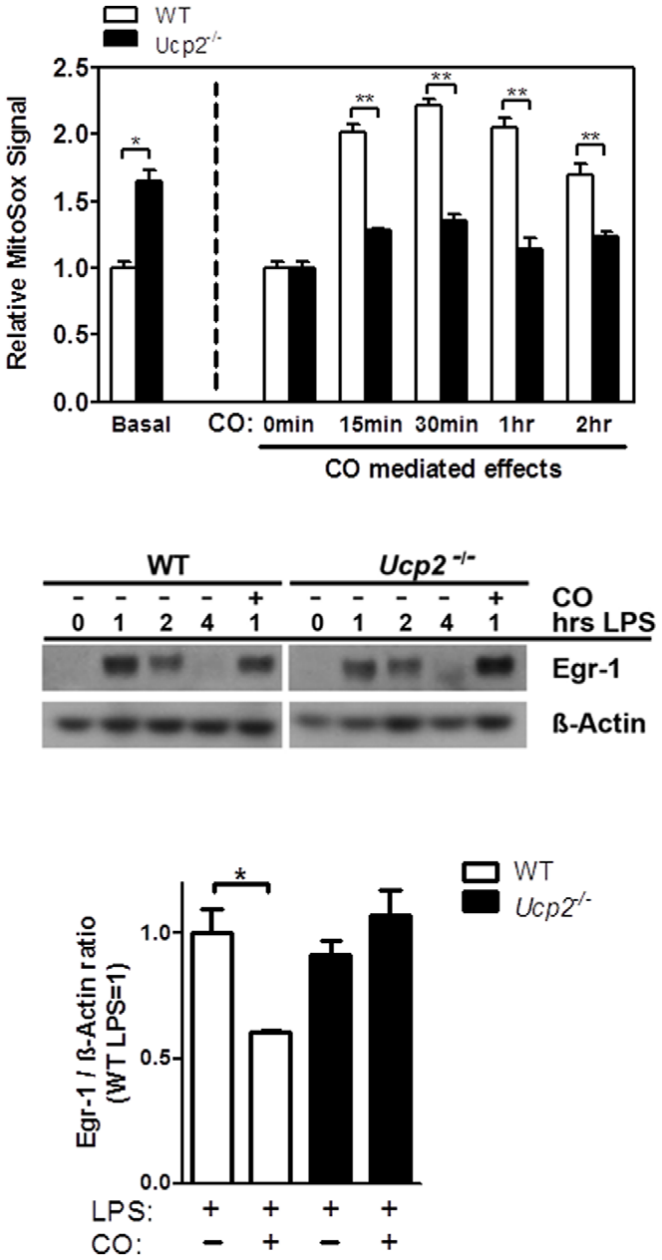
CO increases ROS levels via UCP2 to inhibit Egr-1 expression. (A) Bone-marrow derived macrophages from wild-type (open bars) or *Ucp2* ^−/−^ (filled bars) mice were treated with CO (250 ppm) and ROS formation was measured with MitoSOX at indicated times by FACS analysis. MitoSOX is specific for mitochondria-derived ROS. Left, the basal effect of UCP2 loss was compared to control cells. Right, we show the effect of CO on ROS formation in *Ucp2* ^−/−^ and wild-type macrophages. (C) Bone-marrow derived macrophages from wild-type or *Ucp2* ^−/−^ mice were pretreated with 250 ppm CO or Air for 3 hours, prior to incubation with LPS (10 ng/ml) for indicated times. Egr-1 protein expression was analyzed by western blotting and β-actin was used as a loading control. (D) The bar graph represents densitometry analysis of western blots after 1 hr LPS stimulation in the presence or absence of CO. Depicted western blot is one of three representative experiments. Bar graphs indicate mean fold change ± SEM of three independent experiments (^*^P<0.05 and ^**^P<0.001).

**Figure 5:**
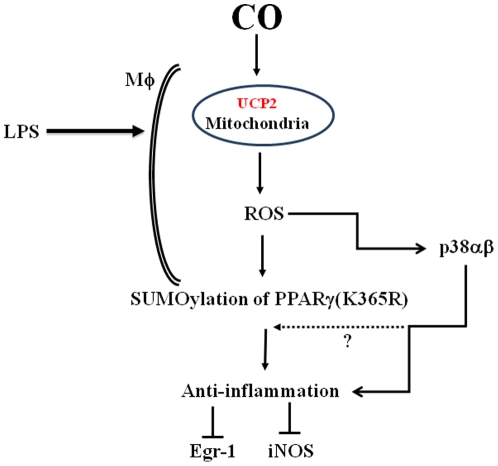
Schematic diagram describing the effects of CO on LPS-activated macrophages. Exposure of macrophages to CO leads to an increase in mitochondria-derived reactive oxygen species (ROS), which in turn drives SUMOylation of PPARγ and activation of p38 mitogen-activated protein kinase. The activation of PPARγ and p38 both contribute in part to modulation of the inflammatory response.

We considered ROS formation by CO as possible key upstream signaling molecule, as ROS mediates both CO-induced p38 activation and PPARγ SUMOylation. CO treatment results in the rapid generation of mitochondrial ROS at complex III of the electron transport chain [18]. Because UCP2 has been implicated in mastering mitochondrial ROS [25], we hypothesized that UCP2 might be involved in the ability of CO to increase ROS. To determine mitochondrial ROS generation, we utilized the mitochondrial ROS detection reagent MitoSOX. This approach specifically detects ROS originating from mitochondria. First, we tested if UCP2 is indeed involved in ROS production by CO exposure and second if CO-induced ROS is mastering Egr-1 repression, a process dependent on p38 activation and PPARγ SUMOylation. Consistent with a report by Arsenijevic et al. [25], we demonstrate here that macrophages from *Ucp2* ^−*/*−^ animals produce higher basal superoxide levels compared to macrophages from wild-type animals (**Fig. 4A** **, Basal**). However, CO exposure of BMDM from wild type mice led to a doubling of MitoSOX Red fluorescence after 15 min, peaking at 30 min and starting to normalize by 2 hrs. *Ucp2* ^−*/*−^ macrophages revealed an ablation of this CO effect and thereby supporting a functional role of UCP2 in promoting CO-induced mitochondrial ROS (**Fig. 4A**). Given that the MitoSOX Red reagent is readily oxidized by superoxide and not by other ROS or reactive nitrogen species (RNS)–generating systems, we conclude that superoxide is the most likely ROS generated by CO. To ultimately test for CO-induced ROS a key trigger in Egr-1 suppression we again employed *Ucp2* ^−*/*−^ BMDM, which we show are deficient in producing ROS by CO. As expected, LPS added to BMDM provoked a strong increase in Egr-1 protein expression in wild-type and *Ucp2* ^−*/*−^ macrophages which peaked 1 hr post stimulation (**Fig. 4B**). CO suppressed LPS-induced Egr-1 expression in wild-type macrophages, as we previously reported, but not in *Ucp2* ^−*/*−^ macrophages (**Fig. 5A**). Densitometric analysis of western blot experiments revealed a total loss of CO mediated effects by blocking UCP2 dependent ROS formation (**Fig. 4C**). Of note, induction of Egr-1 by LPS was not regulated by UCP2. Collectively, these data suggested that CO requires UCP2 to generate mitochondrial ROS and to elicit and impact down stream effects such as Egr-1 repression.

## Discussion

Our most important mechanistic findings in this study include (i) CO treatment resulted in mitochondria driven ROS-dependent SUMOylation of PPARγ and (ii) UCP2 in the mitochondria was in part responsible for ROS generation by CO. Blocking both resulted in nearly a complete loss of CO induced Egr-1 gene-repression. Interestingly, primary *Ucp2* ^−/−^ macrophages confirmed a total loss of CO on ROS formation and consequently on Egr-1 repression – compared to the partial blockade achieved by individual inhibition of p38 and PPARγ-SUMOylation. Blocking both resulted in a complete loss of CO induced Egr-1 gene-repression. It is important to point out that the exact reactive species responsible for the observed CO effects cannot be conclusively identified given that the fluorescence dyes employed are at best selective and not specific. Additionally, using the antioxidant, glutathione precursor, N-AcetylCysteine (NAC), to selectively scavenge ROS, including superoxide and peroxides, we demonstrate that PPARγ-SUMOylation is contingent upon CO-induced ROS formation (**Fig. 1B**). These studies with NAC identified (or confirmed) PPARγ SUMOylation by CO as a process dependent on ROS formation likely superoxide or peroxide. Sugimoto et al. [28] had utilized this strategy to demonstrate ‘proof-of-concept’ increases in the levels of intracellular GSH in THP-1 and Jurkat cell lines. Nevertheless, this series of events explains in part the mechanism by which CO regulates inflammation (**Figure 5**).

The nuclear hormone receptor PPARγ exerts potent anti-inflammatory effects in macrophages [11] and accounts in large part for the anti-inflammatory effects of CO *in vitro* and *in vivo* [1], [9], [10], [26], [27]. Transrepression of inflammatory genes such as iNOS by PPARγ in macrophages depend on SUMOylation at specific amino acid residues of PPARγ [10], [11], [13]. PPARγ contains two possible SUMOylation sites located at K77 and K365. By using PPARγ mutants (i.e. lysine-to-arginine), we demonstrated that SUMOylation of PPARγ by CO is functionally important for the ability of CO to inhibit LPS-induced Egr-1 transactivation in macrophages. These experiments suggested the involvement of K365 rather than K77 SUMOylation as overexpression of PPARγ-K77R mutant failed to perturb Egr-1 repression by CO. This notion is further supported by our finding that CO did not suppress LPS-induced iNOS in PPARγ-K365R mutants, which is in line with previous observations on SUMOylation dependent transrepression of inflammatory gene promoters [11]. However, our studies cannot exclude the possibility that CO increases PPARγ-SUMOylation at K77, but at least this seems not to be sufficient to inhibit Egr-1 expression under our experimental conditions. Because CO acts as a pleiotropic signaling molecule [1], we speculate that CO regulates inflammation, proliferation and apoptosis in part by SUMOylation of other yet-to-be identified protein targets. How exactly CO increases PPARγ SUMOylation remains to be identified, but one likely mechanism may include modification of the SUMO pathway enzymes by ROS [15] originating from mitochondria following exposure of cells to CO [1], [9], [17], [18], [28], [29], [30]. In this context it's important to mention that the SUMO conjugation and deconjugation machinery is highly sensitive to ROS and that increased oxidative stress triggers SUMOylation [31], [32], [33]. Indeed, addition of the ROS scavenger NAC partially blocked PPARγ SUMOylation in response to CO.

The inhibitory effect of CO on LPS-induced Egr-1 gene expression was not completely reversed in the presence of SUMOylation-defective PPARγ mutants. Additional mechanisms may exist including differential regulation of scaffold regulatory corepressors/coactivators such as PGC-1, nuclear receptor corepressor (NcoR), silencing mediator for retinoid and thyroid hormone receptors (SMRT), or phosphorylation by MAP kinases [34]. PPARγ-independent but ROS-dependent mechanisms could include p38MAPK, because (i) anti-inflammatory effects of CO depend on both, ROS and p38 [2], [18], [37] and (ii) we observed a partial loss of CO induced Egr-1 gene-repression upon pharmacological inhibition of p38 MAPK. Future studies shall determine potential links/crosstalks between p38 and PPARγ signaling in the anti-inflammatory actions of CO. Furthermore, there may be synergistic or additive effects of activating both molecules.

Another unresolved issue has been the detailed molecular mechanism underlying increased mitochondrial ROS in response to CO. Suliman et al. [29] reported previously that CO induced UCP2 in H9c2 rat heart cells. In macrophages, CO treatment transiently, but significantly, inhibits cytochrome c oxidase resulting in a hyperpolarization of mitochondrial membrane potential and backup of electrons at complex III of the respiratory chain leading to superoxide production [1], [17], [18]. The physiological function of UCP2 is subject of intense debate as multiple functions have been ascribed to this protein including uncoupling of respiration, as well as Ca^++^ flux [22]. Our findings that CO requires in part UCP2 is in line with reports showing that uncoupling of inhibited mitochondria enhances ROS [22], [35], [36]. How CO modulates UCP2 remains to be identified. Likely candidates include availability of Coenzyme Q, nucleotides, fatty acids in addition to allosteric regulation or phosphorylation of consensus sites within UCP2 by protein kinases [22], [36], [37].

In conclusion, our data continue to expand our understanding as to how CO modulates the remarkable plasticity of macrophages associated with decreased proinflammatory gene expression and restoration of homeostasis. We provide evidence that Ucp2 is involved in CO-increased ROS, which results in PPARγ SUMOylation and p38 hyperphosphorylation, that perhaps act synergistically or additively downstream to block Egr-1 and NOS2 expression (**Figure 5**). CO, which is currently in clinical trials as an inhaled therapeutic, may evolve as an important homeostatic molecule in the treatment of inflammatory disorders.
